# Patterns of Use and Outcomes in Patients Treated with Etravirine in the HIV Research Network

**DOI:** 10.1155/2013/492831

**Published:** 2013-01-29

**Authors:** Kelly Gebo, Cindy Voss, Joseph Mrus, HIV Research Network

**Affiliations:** ^1^School of Medicine, Johns Hopkins University, 1830 East Monument Street Suite 435, Baltimore, MD 21287, USA; ^2^Janssen Services, LLC, Titusville, NJ 08560, USA

## Abstract

This observational analysis examined the clinical outcomes of patients receiving etravirine-(ETR-) based therapy, particularly with protease inhibitors (PIs) other than darunavir (DRV) and with raltegravir (RAL). Data included treatment-experienced adults in the HIV Research Network who began ETR-containing antiretroviral regimens in 2008–2010. The primary objective was to assess 6-month outcomes (durability, i.e., still on an ETR-containing regimen; change in CD4+ cell count and HIV-1 RNA <400 copies/mL). The cohort included 587 patients receiving ETR; 42% of ETR use was in patients not on DRV/ritonavir (r). Patients receiving ETR plus DRV/r had longer durability versus those on ETR plus a PI other than DRV/r at months 6 (91.2% versus 85.5%) and 12 (77.4% versus 65.2%), respectively. Patients on regimens with a PI other than DRV/r were the least likely to be receiving ETR at month 6 (85.5%) versus patients on other ETR-based regimens. Patients on regimens without a PI and without RAL had lower virologic suppression (month 6, 54.2%; month 12, 63.2%) versus patients on other ETR-based regimens. In a clinical care, nontrial setting, ETR was used in regimens without DRV/r. In this population, the 6-month response rates were similar and durable across all regimens, except when ETR was used without RAL and without a PI.

## 1. Introduction 

Aside from registrational trial data, there is limited information on the utilization and clinical outcomes of patients treated with etravirine-(ETR-) based therapy. To date, ETR has demonstrated high efficacy rates, as well as good tolerability and safety profiles [1, 2]. However, in most studies of ETR, including the Phase III trials, all patients also received darunavir/ritonavir (DRV/r). In the DUET 1 and DUET 2 trials, for example, 61% of patients receiving ETR 200 mg twice daily (bid) plus a background regimen that included DRV/r 600/100 mg bid achieved HIV-1 RNA <50 copies/mL at 48 weeks, compared with 40% of patients receiving placebo plus a background regimen that included DRV/r 600/100 mg bid [2]. Interestingly, in the GRACE (Gender, Race, And Clinical Experience) trial, which investigated the efficacy and safety of DRV/r plus an optimized background regimen that could include ETR in treatment-experienced patients, ETR use was associated with higher virologic response rates [3].

Despite encouraging data on the use of ETR plus DRV/r, limited data exist on the use of ETR with protease inhibitors (PIs) other than DRV/r, and with novel agents like raltegravir (RAL) [1, 4–6]. The few data that do exist are promising. The Phase II Agence Nationale de Recherches sur le SIDA et les hépatites virales (ANRS) 139 TRIO trial demonstrated that a regimen of RAL 400 mg bid plus ETR 200 mg bid and DRV/r 600/100 mg bid was highly efficacious, with 88% of patients achieving HIV-1 RNA <50 copies/mL at 96 weeks [1]. Additionally, in a small study of 28 treatment-experienced patients receiving maraviroc, RAL, and ETR, 96% of patients achieved virologic response at 96 weeks [4].

We evaluated outcomes with the use of ETR in a consortium of high-volume HIV care sites to describe the current utilization of antiretroviral therapy. 

## 2. Materials and Methods

### 2.1. Data Collection and Patients

We analyzed antiretroviral medication utilization among HIV-infected adults enrolled in the HIV Research Network (HIVRN), a consortium of clinics that provide primary and subspecialty care to HIV patients. Fifteen sites treat adult patients. Data from 12 sites, located in the northeastern (5), midwestern (1), southern (3), and western (3) United States, were included in this analysis. The remaining three sites discontinued participation during the study period and did not provide complete data. Nine sites have academic affiliations. Adult patients (≥18 years of age) who enrolled at an HIVRN site between 2000 and 2010 and who had at least one outpatient visit and a CD4 count in any calendar year between 2000 and 2010 were eligible for inclusion. 

Data encompassing the period from January 1, 2008, through December 31, 2010, were gathered from medical records at each site and sent to a data coordinating center after personal identifying information was removed. Problematic data elements were identified, reviewed with the site, and corrected. After quality control and verification, data were combined across sites to produce a uniform database. The study was approved by institutional review boards at the Johns Hopkins University School of Medicine and at each participating site.

Over 20,000 patients are represented at 12 sites. Of these, 29% are women, 51% are black, 22% are Hispanic, 51% are receiving Medicare and/or Medicaid, 18% are receiving commercial insurance, and 24% are receiving Ryan White services (a federal program providing HIV-related services to those who do not have sufficient health care coverage or financial resources). Here, we present data collected from the HIVRN on the outcomes of patients receiving a variety of ETR-based therapies over 6 and 12 months.

Demographic and clinical data were collected from treatment-experienced adult (≥18 years of age) patients in the HIVRN who began an ETR-containing antiretroviral regimen in 2008 or 2009. Demographic data for treatment-experienced adult patients in the HIVRN who began an antiretroviral regimen not containing ETR in 2008, 2009, or 2010 are also included for comparison. To be included, ETR had to be prescribed for at least 60 days and patients had to have at least 24 weeks of follow-up after beginning the regimen. 

### 2.2. Study Outcomes

The primary objective was to assess 6-month outcomes (durability, change in CD4 cell count, and observed HIV-1 RNA <400 copies/mL). Outcomes are reported by regimen up to 12 months. As not all HIV-1 RNA tests performed in routine care had a lower limit of quantification of <50 copies/mL, HIV-1 RNA <400 copies/mL was chosen as the primary end point and considered suppression for this study.

### 2.3. Statistical Evaluation

Sample size was based on convenience and represented all available patients who met inclusion criteria in the cohort. As the sample was based on convenience and the study was not powered for between-group comparisons, only descriptive statistics are presented. Analyses were performed using SAS 9.1.3 (SAS Institute Inc., Cary, NC). 

## 3. Results

### 3.1. Patient Population and Baseline Demographics

In total, 587 patients on ETR-containing regimens met inclusion criteria and were included in the cohort. Compared with the 15,084 patients from the HIVRN who were treatment experienced and did not receive ETR, the patients from this cohort were older, had lower median baseline CD4+ cell counts and CD4+ nadir counts, and had been in care for a longer period (Table 1).

In this cohort, 42% of the ETR use was by patients not on DRV/r, and 46% of ETR-based regimens included both a PI and RAL (Table 1). Of the patients who received a PI other than DRV/r (*n* = 69), 27 (39.1%) received lopinavir/r, 17 (24.6%) received atazanavir, and 4 (5.8%) received fosamprenavir. 

At baseline, the group of patients treated with ETR without a PI and those treated with ETR without a PI and without RAL had a lower proportion of male and white patients, a higher proportion of Hispanic patients, a shorter median time in care, and a high median nadir CD4 count compared with patients on other regimens (Table 1). Patients treated with ETR in addition to a PI and RAL were more frequently white and had the lowest nadir CD4+ cell count compared with patients on other regimens (Table 1). 

Patients treated with ETR and DRV/r were more frequently male and had lower median nadir CD4+ cell counts and a lower incidence of injection drug use compared with those treated with ETR and other PIs or those treated with ETR and no PI (Table 1). 

### 3.2. Six- and Twelve-Month Outcomes by Regimen

**Figure 1:**
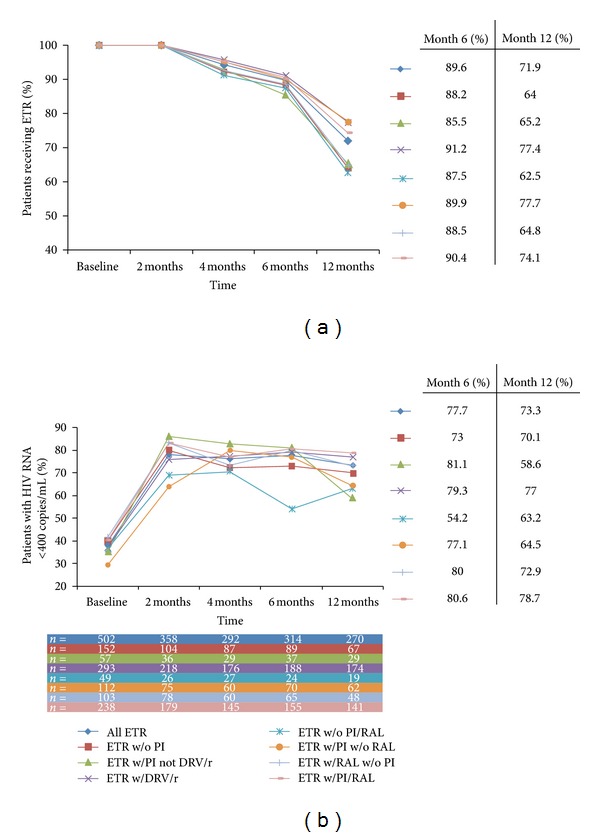
Proportion of patients (a) receiving etravirine over time, by regimen and (b) with HIV-1 RNA <400 copies/mL over time (observed), by regimen. ETR: etravirine; w/o: without; PI: protease inhibitor; w/: with; DRV/r: darunavir/ritonavir; RAL: raltegravir.

Patients treated with ETR plus DRV/r had higher durability (i.e., were still on ETR) than those treated with ETR plus a PI other than DRV/r at month 6 and at month 12 (Table 1, Figure 1(a)). Patients on regimens with a PI other than DRV/r were the least likely to still be receiving ETR at month 6 and those treated with ETR without a PI and without RAL were the least likely to still be receiving ETR at month 12 compared with patients on other ETR-based regimens (Table 1, Figure 1(b)).

Rates of viral suppression (<400 copies/mL) were similar for patients receiving ETR plus DRV/r compared with those receiving ETR plus a PI other than DRV/r through month 6, whereas rates of viral suppression at 12 months were higher in patients receiving ETR plus DRV/r compared with those receiving ETR plus a PI other than DRV/r (Table 1, Figure 1(b)). Patients treated with ETR plus RAL either with or without a PI had higher rates of HIV-1 RNA suppression compared with those treated with ETR without RAL either with or without a PI, at month 6 and at month 12 (Table 1, Figure 1(b)). 

Patients on regimens without a PI and without RAL had lower rates of HIV-1 RNA suppression compared with patients on other ETR-based regimens at month 6 and at month 12 (Table 1, Figure 1(b)). In contrast, these patients also demonstrated the largest median CD4+ cell count increase from baseline to month 6 (Table 1), compared with other ETR-based regimens. 

## 4. Discussion

The registrational studies for ETR all included DRV/r as part of the treatment regimen [2]. However, 42% of the patients in this cohort received ETR as part of a DRV/r-free regimen, indicating that patients in a clinical care, non-trial setting often receive ETR without DRV/r. Virologic suppression rates appeared similar when ETR was used with DRV/r or other PIs through 6 months. Interestingly, however, the durability was higher for patients receiving an ETR-DRV/r–based regimen compared with that of patients receiving ETR plus a PI other than DRV/r. Suppression rates for patients receiving ETR plus DRV/r were higher in this analysis at month 12 (77.0%) than for those receiving ETR plus another PI (58.6%). Similarly, in the intent-to-treat analysis of the pooled DUET trials at week 48, 72% of patients receiving ETR, DRV/r, and other background antiretrovirals achieved HIV-1 RNA <400 copies/mL [2].

Suppression rates at month 6 and month 12 were lower when ETR was combined with neither a PI nor RAL. Suppression at month 6 was similar between patients treated with ETR plus RAL (80.0%) and those treated with ETR plus RAL and a PI (80.6%). These results suggest that, when deemed appropriate based on patient history and resistance testing, an ETR plus RAL-based regimen may be a viable treatment option. Although still high, the suppression rates in patients receiving ETR with a PI and RAL (month 6, 80.6%; month 12, 78.7%) in this trial were slightly lower than those seen in the TRIO study (88%) [1]. This inconsistency is likely due to the difference in study design (cohort versus clinical trial), patient populations (real-world versus study), and inclusion criteria (not specified versus required susceptibility to DRV and/or ETR). Given the highly treatment-experienced nature of the patient population, the response rates in this cohort are high and suggest that ETR-based regimens are efficacious in this population.

Several limitations to this analysis should be noted. First, there was only a small population of patients receiving ETR in the HIVRN. Second, the differences in baseline characteristics of patients between the different treatment regimens were likely due to providers prescribing the different regimens based on perceived needs of the patients that included some of the observed characteristics as well as other characteristics not noted in this study (i.e., baseline resistance). Adjustment for these variations in baseline characteristics by PI and RAL use was not performed in this analysis. Finally, the potential antiviral activity of ETR and other antiretrovirals was unknown before treatment initiation, as resistance testing results before starting ETR were not available.

## 5. Conclusions

Results from this observational analysis demonstrated that, in a real-world setting, ETR was used in regimens not containing DRV/r and that in a highly treatment-experienced patient population, the response rates at 6 months were similar and durable across all combinations studied—except when ETR was used without RAL or a boosted PI. Future studies will need to explore whether certain populations may be more likely to benefit from one regimen compared with another. 

## Figures and Tables

**Table 1:** Baseline demographics and disease characteristics by regimen.

	Treatment-experienced, non-ETR (*N* = 15,084)^a^	All ETR (*N* = 587)	ETR w/o PI (*n* = 178)	ETR w/PI not DRV/r (*n* = 69)	ETR w/DRV/r (*n* = 340)	ETR w/o PI/RAL (*n* = 56)	ETR w/PI w/o RAL (*n* = 139)	ETR w/RAL w/o PI (*n* = 122)	ETR w/PI/RAL (*n* = 270)
Baseline characteristics									
Male, *n* (%)	10,667 (70.7)	434 (73.9)	107 (60.1)	47 (68.1)	280 (82.4)	30 (53.6)	107 (77.0)	77 (63.1)	220 (81.5)
Median (range) age at start of ETR regimen, years	44 (18, 90)	47 (18, 86)	48 (22, 86)	45 (18, 63)	46 (21, 79)	46 (22, 78)	46 (18, 79)	49 (24, 86)	46 (21, 72)
Race, *n* (%)									
White	3858 (25.6)	144 (24.5)	35 (19.7)	15 (21.7)	94 (27.7)	10 (17.9)	29 (20.9)	25 (20.5)	80 (29.6)
Black	7581(50.3)	291 (49.6)	85 (47.8)	37 (53.6)	169 (49.7)	21 (37.5)	71 (51.1)	64 (52.5)	135 (50.0)
Hispanic	3272 (21.7)	140 (23.9)	53 (29.8)	16 (23.2)	71 (20.9)	23 (41.1)	37 (26.6)	30 (24.6)	50 (18.5)
Other	373 (2.5)	12 (2.0)	5 (2.8)	1 (1.4)	6 (1.8)	2 (3.6)	2 (1.4)	3 (2.5)	5 (1.9)
IDU, *n* (%)	2614 (17.3)	102 (17.4)	36 (20.2)	14 (20.3)	52 (15.3)	13 (23.2)	23 (16.6)	23 (18.9)	43 (15.9)
Median (range) years in care	4 (0, 22)	7 (0, 22)	6 (0, 19)	8 (0, 22)	7 (0, 20)	4.5 (0, 19)	7 (0, 19)	7 (0, 19)	8 (0, 22)
Median (range) CD4+ nadir, cells/mL	170 (0, 1802) *n* = 14,334	57 (0, 1260) *n* = 572	104 (0, 749)	72.5 (0, 594)	55 (0, 1260)	200 (6, 708)	73 (0, 1260)	70.5 (0, 749)	44 (0, 720)
Median (range) baseline CD4+ cell count, cells/mL	322 (0, 3167) *n* = 12,059	240 (0, 1187) *n* = 501	242.5 (2, 1134)	232 (4, 770)	244 (0, 1187)	292 (7, 1031)	258.5 (3, 899)	201 (2, 1134)	228 (0, 1187)

Six-month outcomes									
Durability (still on ETR), *n* (%)	NA	526 (89.6)	157 (88.2)	59 (85.5)	310 (91.2)	49 (87.5)	125 (89.9)	108 (88.5)	244 (90.4)
Median (range) change in CD4+ cell count (observed), cells/mL	NA	65 (−483, 570)	76 (−341, 361)	89 (−132, 527)	62 (−483, 570)	103.5 (−123, 361)	24.5 (−157, 276)	49 (−341, 278)	74 (−483, 570)
Suppressed HIV-1 RNA (<400 copies/mL; observed), *n*/*n* (%)	NA	244/314 (77.7)	65/89 (73.0)	30/37 (81.1)	149/188 (79.3)	13/24 (54.2)	54/70 (77.1)	52/65 (80.0)	125/155 (80.6)
Suppressed HIV-1 RNA (<50 copies/mL; observed), *n*/*n* (%)	NA	161/314 (51.3)	45/89 (50.6)	13/37 (35.1)	103/188 (54.8)	8/24 (33.3)	38/70 (54.3)	37/65 (56.9)	78/155 (50.3)

^a^All treatment-experienced patients from the HIVRN who did not receive ETR; ETR: etravirine; w/o: without; PI: protease inhibitor; w/: with; DRV/r: darunavir and ritonavir; RAL: raltegravir; PI/RAL: protease inhibitor and raltegravir; IDU: injection drug use; NA: not applicable; VL: viral load; HIVRN: HIV Research Network.

## References

[1] Fagard C, Descamps D, Colin C Long term follow-up of patients receiving raltegravir, etravirine and darunavir/ritonavir in the ANRS 139 TRIO trial.

[2] Katlama C, Haubrich R, Lalezari J (2009). Efficacy and safety of etravirine in treatment-experienced, HIV-1 patients: pooled 48 week analysis of two randomized, controlled trials. *AIDS*.

[3] Kumar P, Currier J, Squires K, Mrus J, Coate B, Ryan R Predictors of response in GRACE (Gender, Race And Clinical Experience).

[4] Nozza S, Galli L, Visco F (2010). Raltegravir, maraviroc, etravirine: an effective protease inhibitor and nucleoside reverse transcriptase inhibitor-sparing regimen for salvage therapy in HIV-infected patients with triple-class experience. *AIDS*.

[5] Skiest DJ, Cohen C, Mounzer K (2011). Similar efficacy of raltegravir when used with or without a protease inhibitor in treatment-experienced patients. *HIV Clinical Trials*.

[6] Towner W, Lalezari J, Sension MG (2010). Efficacy, safety, and tolerability of etravirine with and without darunavir/ritonavir or raltegravir in treatment-experienced patients: Analysis of the etravirine early access program in the United States. *Journal of Acquired Immune Deficiency Syndromes*.

